# Combined proteomic/transcriptomic signature of recurrence post-liver transplantation for hepatocellular carcinoma beyond Milan

**DOI:** 10.1186/s12014-021-09333-x

**Published:** 2021-11-18

**Authors:** Mamatha Bhat, Sergi Clotet-Freixas, Cristina Baciu, Elisa Pasini, Ahmed Hammad, Tommy Ivanics, Shelby Reid, Amirhossein Azhie, Marc Angeli, Anand Ghanekar, Sandra Fischer, Gonzalo Sapisochin, Ana Konvalinka

**Affiliations:** 1 Ajmera Transplant Program, University Health Network, Toronto, Canada; 2 Division of Gastroenterology and Hepatology, University of Toronto, Toronto, Canada; 3 Toronto General Hospital Research Institute, Toronto, Canada; 4 Department of General Surgery, Mansoura University, Mansoura, Egypt; 5 Department of Laboratory Medicine and Pathology, University of Toronto, Toronto, Canada; 6 Division of Multi-Organ Transplant and HPB Surgical Oncology, Department of General Surgery, University Health Network, Toronto, Canada; 7 Institute of Medical Science, University of Toronto, Toronto, Canada; 8 Division of Nephrology, Department of Medicine, University Health Network, Toronto, Canada; 9 University Health Network, 585 University Avenue, Room 11-PMB-189, Toronto, ON M5G 2N2 Canada

**Keywords:** Hepatocellular carcinoma, Liver transplantation, Milan criteria, Proteome, Transcriptome, Combined signature, Tumor explant, Predictors of recurrence

## Abstract

**Background and aims:**

Liver transplantation (LT) can be offered to patients with Hepatocellular carcinoma (HCC) beyond Milan criteria. However, there are currently limited molecular markers on HCC explant histology to predict recurrence, which arises in up to 20% of LT recipients. The goal of our study was to derive a combined proteomic/transcriptomic signature on HCC explant predictive of recurrence post-transplant using unbiased, high-throughput approaches.

**Methods:**

Patients who received a LT for HCC beyond Milan criteria in the context of hepatitis B cirrhosis were identified. Tumor explants from patients with post-transplant HCC recurrence (N = 7) versus those without recurrence (N = 4) were analyzed by mass spectrometry and gene expression array. Univariate analysis was used to generate a combined proteomic/transcriptomic signature linked to recurrence. Significantly predictive genes and proteins were verified and internally validated by immunoblotting and immunohistochemistry.

**Results:**

Seventy-nine proteins and 636 genes were significantly differentially expressed in HCC tumors with subsequent recurrence (p < 0.05). Univariate survival analysis identified Aldehyde Dehydrogenase 1 Family Member A1 (ALDH1A1) gene (HR = 0.084, 95%CI 0.01–0.68, p = 0.0152), ALDH1A1 protein (HR = 0.039, 95%CI 0.16–0.91, p = 0.03), Galectin 3 Binding Protein (LGALS3BP) gene (HR = 7.14, 95%CI 1.20–432.96, p = 0.03), LGALS3BP protein (HR = 2.6, 95%CI 1.1–6.1, p = 0.036), Galectin 3 (LGALS3) gene (HR = 2.89, 95%CI 1.01–8.3, p = 0.049) and LGALS3 protein (HR = 2.6, 95%CI 1.2–5.5, p = 0.015) as key dysregulated analytes in recurrent HCC. In concordance with our proteome findings, HCC recurrence was linked to decreased ALDH1A1 and increased LGALS3 protein expression by Western Blot. LGALS3BP protein expression was validated in 29 independent HCC samples.

**Conclusions:**

Significantly increased LGALS3 and LGALS3BP gene and protein expression on explant were associated with post-transplant recurrence, whereas increased ALDH1A1 was associated with absence of recurrence in patients transplanted for HCC beyond Milan criteria. This combined proteomic/transcriptomic signature could help in predicting HCC recurrence risk and guide post-transplant surveillance.

**Supplementary Information:**

The online version contains supplementary material available at 10.1186/s12014-021-09333-x.

## Introduction

Hepatocellular carcinoma (HCC) is estimated to represent the fourth most common cause of cancer-related deaths globally [1]. Selected patients with early-stage HCC are candidates for liver transplantation (LT) as a curative therapy. HCC has even become the leading indication for LT worldwide in recent years [1, 2]. Currently, the Milan criteria (one lesion less than 5 cm in diameter or three or fewer lesions, all below 3 cm) remain the gold standard for defining the optimum tumor burden that determines transplant eligibility [3]. Nonetheless, it has been recognized that expanded selection criteria can afford patients excellent post-LT outcomes even when traditional morphologic tumor standards are exceeded [4]. Criteria have evolved by expanding the allowable tumor size and number to the incorporation of alpha-fetoprotein (AFP) and histologic differentiation [4–9]. The safe expansion of selection criteria for LT in HCC hinges on an improved understanding of tumor biology through surrogate markers [1]. Consequently, such expanded criteria may allow extending the use of LT as a curative-intent treatment option to a greater number of patients, especially considering that HCC biology and progression are patient-dependent.

Numerous studies have tried to identify molecular biomarkers that predict HCC recurrence after LT or resection [10–18]. In this context, proteomic analysis of liver tissue from HCC patients has recently identified some proteins such as melanoma-associated antigen genes [19] and cytokeratin-19 [20]; their expression correlated with early recurrence of HCC post-hepatectomy and allowed stratification into various subtypes with distinct clinical outcomes [11]. Whether such proteomic signatures can stratify patients in a diverse HCC LT population that exceeds Milan criteria is unclear. Identifying novel molecular signatures may enable the selection of patients exceeding traditional standards who may still benefit from LT. Also, signature proteins related to distinct tumor subtypes may potentially serve as actionable targets for individualized prevention and therapy.

We hypothesized that genes and proteins shared among HCC tumors on explant could be used to predict recurrence. Therefore, the goal of the present study was to define a combined proteomic/transcriptomic signature predictive of recurrence post-transplant in patients transplanted for HCC beyond Milan criteria, based on the molecular profiling of the dominant tumour on explant.

## Methods

### Patient population

Patients who underwent LT for hepatitis B-induced HCC beyond Milan criteria between 2004 and 2015 were included in our study. This protocol was approved by the University Health Network (UHN) Institutional Review Board (REB#15-9989). Those with recurrence were matched for age and sex to those without recurrence by at least 2 years post-transplant. The first set of patients (n = 11) had snap-frozen HCC explant samples in the biobank, meeting inclusion criteria, and was therefore eligible for proteome and transcriptome analysis. An additional set of 29 samples meeting the above inclusion criteria were from patients with only archived formalin-fixed, paraffin-embedded samples available, on which only immunohistochemistry (IHC) validation was feasible. Characteristics of the tumors on explant, including size and number of tumors, microvascular invasion and associated AFP at the time of transplant were documented. Patients could have received bridging therapy but needed to have a viable tumor on the explant for the purposes of this analysis.

### Hepatocellular carcinoma sample preparation for mass spectrometry

Frozen HCC tissue samples obtained from the UHN biobank were processed and analyzed in a blinded fashion. These represented the largest, viable tumor in the explant. Rapigest detergent (0.2%) was added to each piece of tissue (~ 100 mg in weight). Samples were then homogenized using microbeads in a tissue dissociator. Ammonium bicarbonate was added, and samples were subjected to sonication (10 s, 3 times) on ice. Samples were then centrifuged at 15,000*g* at 4 °C for 20 min. Supernatant was collected and vortexed. Total protein concentration was determined using the micro-BCA protein assay kit (Thermo). Each sample was then normalized to 250 µg of total protein. Samples were denatured at 80 °C for 15 min, followed by reduction in 10 mM dithiothreitol (final concentration) for 15 min at 65 °C and alkylation in 20 mM (final concentration) iodoacetamide for 40 min in the dark at room temperature. Finally, trypsin (Promega) was added at 1:50 w/w and incubated overnight at 37 °C. Digested samples were acidified with trifluoroacetic acid (1% v/v) and vortexed for 1 min, then left at room temperature for 5 min. Samples were centrifuged at 15,000*g* for 10 min to remove Rapigest, then transferred into the new tubes and frozen at – 20 °C until further analysis [21, 22]. Following digestion and removal of Rapigest, strong cation exchange (SCX) chromatography and fractionation were performed on an HPLC system (Agilent 1100) using a 60-min two-step gradient. The resulting fractions corresponding to chromatographic peaks of eluting peptides were pooled into 10 fractions. Peptides from each fraction were extracted, desalted and diluted to 41 μL with 0.1% v/v formic acid in MS-grade pure water.

### Tandem mass spectrometry (MS/MS)

Samples were randomized and subjected to mass spectrometry (MS) on a Thermo Scientific EASY-nLC1000 system, coupled to a Q-Exactive Plus hybrid quadrupole-orbitrap mass spectrometer using a nano-electrospray ionization source (Thermo Scientific) [21–23]. For each HCC sample, 18 μL of eluted peptides were injected onto a 3.3 cm C18 pre-analytical column (IntegraFrit capillary, New Objective; inner diameter: 75 μm; bead size: 5 μm; Agilent Technologies) followed by a C18 resolving analytical column (PicoTip emitter, inner diameter: 15 cm × 75 μm; tip: 8 μm tip; bead size: 3 μm; Agilent Technologies). Samples were run on a 60-min gradient of increasing concentrations of Buffer B (100% acetonitrile) in 0.1% formic acid/99.9% MS grade water (Thermo Scientific). The method started at 1% Buffer B, and the concentration was increased to 5% at 2 min, with increases to 35% (49 min), 65% (52 min) and 100% (53 min). The spectra were obtained under data-dependent acquisition mode, consisting of full MS1 scans (m/z range: 400–1500; resolution: 70,000) followed by MS2 scans of the top 15 parent ions (resolution: 17,500).

### Protein identification and quantification

For protein identification, the RAW files of each MS run were generated by XCalibur software v3.0.63 (Thermo Scientific). Raw data were analyzed by MaxQuant software (version 1.5.3.28) and searched against the human Uniprot FASTA database (HUMAN5640_sProt–072016, update of July 20, 2016). Proteins and peptides were identified with a false discovery rate of 1%. A minimum length of 6 amino acids was selected. The false positive rate was determined using reversed mode. Trypsin/P was selected as digestion enzyme, and a maximum of 2 missed cleavages was enabled. While cysteine carbamidomethylation was selected as a fixed modification, methionine oxidation and N-terminal acetylation were set as variable modifications. The initial peptide tolerance against a ‘human-first-search’ database was set to 20 ppm. The main search peptide mass tolerance was 40 ppm, and the fragment mass MS/MS tolerance was 0.5 Da. Matching between runs was selected. Normalized label-free quantification (LFQ) of proteins was derived from extracted ion current information from razor and unique peptides with a minimum ratio count of 1. The mass spectrometry proteomics data have been deposited to the ProteomeXchange Consortium via the PRIDE [24] partner repository with the dataset identifier PXD022881 (Reviewer account details: Username: reviewer_pxd022881@ebi.ac.uk; Password: 2GmXJTJo).

Proteomics data were analyzed using Perseus software (version 1.5.2.6). Reverse hits and contaminants were manually checked and removed. Distribution of log2-transformed LFQ intensity values of all quantified proteins was examined for each sample (Additional file 1: Fig. S1A). Following exclusion of proteins identified in < 50% of the samples, we subjected the zero-value intensities to imputation (assuming that low abundance values were missing), keeping a normal distribution, with a downshift of 1.8 standard deviations, and a width of 0.5 for each sample (Additional file 1: Fig. S1B). After imputation, we determined the differentially expressed proteins between recurrent and non-recurrent HCC samples by comparing their mean log2-transformed LFQ intensities using the two-tailed independent t-test (P < 0.05), followed by Benjamini–Hochberg adjustment. Principal component analysis was performed in Perseus. Two components that explained the most variability in samples were selected. Finally, pathway analysis was performed on significantly differentially expressed proteins, using pathDIP (http://ophid.utoronto.ca/pathDIP/) [25].

### Gene expression analysis

RNA was extracted from snap-frozen HCC explant tumor specimens and purified using RNeasy Mini Kit (Qiagen; Hilden, Germany). RNA quality was verified by Nanodrop spectrophotometer (VWR; Radnor, PA) and Bioanalyzer (Agilent; Santa Clara, CA). Gene expression microarray analysis was performed using the Affymetrix Human Gene 2.0 ST platform (Thermo Fisher, Waltham, MA). The initial cohort included eleven samples, but two of them did not pass the quality control filter required for microarray processing, therefore the remaining nine were used for further analysis. We compared gene expression of the explant HCC specimens between those who developed recurrence (n = 6) versus those who did not develop recurrence (n = 3).

Microarray data were pre-processed (background subtraction and quantile normalization) by Robust Multi-array Average (RMA) utilizing the oligo package in R version 3.6 [26]. Gene annotation was done using pd.hugene.2.0.st annotation files [27]. Statistical analysis was performed with limma package [28]. After having fit the model with lmFit function (linear model), the differential gene expression was calculated using eBayes function (moderated t-test, p-value, B stats). A gene was considered differentially expressed between the two groups if p-value < 0.05. In addition, filtering criteria by fold change (FC) was selected: FC ≥ 2 (upregulation) or FC ≤ 0.5 (downregulation).

For identification of protein–protein interactions and pathways in explant HCC that were predictive of HCC recurrence, we analyzed the common significant genes/proteins separately for the two groups using STRING software, version 10.5 (https://string-db.org). The transcriptomic data have been deposited to Gene Expression Ominibus (GEO) repository with the dataset identifier GSE164368.

### Protein expression analysis by Western Blot

For validation purposes, we selected only those proteins in common with corresponding differentially expressed genes, namely Galectin 3 (LGALS3), Galectin 3 Binding Protein (LGALS3BP), and Aldehyde Dehydrogenase 1 Family Member A1 (ALDH1A1), that were significantly associated with the time of recurrence by univariate analysis, as described in the statistical analysis section.

We measured protein expression by Western blot in the same human HCC tissue samples used for proteome analysis. Protein concentration was determined using a micro-BCA protein assay kit (Thermo). To verify changes in LGALS3, LGALS3B and ALDH1A1 protein expression, 10 µg of total protein were loaded onto 10% acrylamide gels, separated by SDS-PAGE, and transferred to a PVDF membrane (Millipore). Membranes were then blocked with 5% milk and incubated with mouse monoclonal anti-LGALS3 (1:4000; ab2785, Abcam, previously characterized [29]) or rabbit polyclonal anti-ALDH1A1 (1:2000; ab227948, Abcam [30]). Incubation with rabbit polyclonal anti-LGALS3BP (HPA000554, Atlas Antibodies) did not result in a specific band. Control for protein loading was performed by reblotting membranes using a mouse monoclonal antibody for GAPDH (CB1001, Sigma). The secondary antibodies were HRP-conjugated anti-rabbit (A0545, Sigma) and anti-mouse (P0447, Dako). Following detection in a Gel-Imaging System (Bio-rad), bands were quantified by densitometry using Image J software.

### Immunohistochemistry

Protein expression of LGALS3BP was examined by immunohistochemistry on the first set of patients (n = 11), with snap-frozen samples in the biobank meeting inclusion criteria (Table 1). LGALS3BP protein expression was also studied in an additional, independent set of 29 patients (Additional file 8: Table S6). Sections (4 µm) from formalin-fixed paraffin-embedded samples were treated for antigen retrieval following manufacturer’s instructions. Rabbit anti-LGALS3BP (Human Protein Atlas, HPA000554) was used at a dilution of 1:500 as primary antibody. After incubation with an HRP-conjugated anti-rabbit secondary antibody, the antibody-HRP complex was visualized with hydrogen peroxide substrate and 3,3′-diaminobenzidine tetrahydrochloride (DAB) chromogen. Slides were then counterstained with Harris Hematoxylin. Entire slides were then digitally scanned in an Aperio ScanScope CS scanning system and analyzed by Aperio Image Scope Viewer software (Leica Biosystems Imaging, Inc, CA) using the Positive Pixel Count v9 algorithm. The intensity of the strongly positive pixels was quantified in each area and normalized to the µm^2^ of area analyzed. The strongly positive intensity/µm^2^ across all areas were then averaged to determine LGALS3BP protein expression in each HCC sample.

**Table 1:** Clinical characteristics of the patients transplanted for HCC beyond Milan criteria with versus without recurrence post-transplant

Variable	Non-recurrent group (n = 4)	Recurrent group (n = 7)	*P* value
Preoperative factors			
Mean age (range), y	64 (58–71)	55 (46–61)	***0.009***
Sex, no. of men (%)	4 (100)	6 (85.7)	1.000
Race, n (%)			0.730
Caucasian	0	1	
Asian	3	5	
Black			
Hispanic			
Native American			
Other/unreported	1	1	
Clinical CPT score before transplant, *n* (%)			0.491
A	4	5	
B	0	2	
C	0	0	
Clinical MELD score at transplant, median (range)	9 (8–9)	9 (8–13)	0.323
Bridging/downstaging LRT			0.509
1. SIRT	0	0	
2. RFA	3	2	
3. TACE	0	3	
4. PEIT	1	1	
5. Combined	0	0	
AFP, median (range), at the time of transplant (μmol/L)	3 (3–48)	68 (5–20,303)	0.453
Waiting list time (time to LT), median (range), months	4 (2–5)	5 (2–7)	0.407
Intraoperative factors			
Cold ischemia time, median (range), minutes	327 (301–470)	421 (275–761)	0.212
Warm ischemia time, median (range), minutes	44 (42–55)	44 (37–53)	0.414
Estimated blood loss, median (range), mL	1070 (230–2100)	1500 (1000–2800)	0.253
Pathologic factors			
Weight of explanted liver, median (range), (g)	766 (750–1650)	1005 (936–1059)	0.804
Largest tumor diameter (cm), mean ± SD (range)	3.5 ± 0.6	4.6 ± 1.8	0.156
Number of lesions on explant, median (range)	5 (5–9)	7 (1–20)	0.323
Capsular invasion (yes), n (%)	0	3 (43%)	0.236
Satellite nodules, (yes) (%)	0	2 (29%)	0.491
HCC histologic grade			1.000
Moderately differentiated	4 (100%)	6 (86%)	
Well-differentiated	0	1 (14%)	
Presence of microvascular invasion on the explant (%)	1 (25%)	4 (57%)	0.546
Major vessel invasion	0	1 (14%)	1.000
Bile duct invasion	0	0	1.000
Portal vein invasion	0	0	1.000
HCC-related outcome			
Vital status			0.109
Alive	2	0	
Dead	2	7	
Overall survival, (years)	6 (0–11)	3 (1.2–7.5)	0.261
Time to recurrence, (months)	N/A	12 (3–24)	

Count and median values are reported, with the 95% confidence intervals displayed in the brackets

Significant *P* value is in bold italic

*AFP* alpha fetoprotein, *CPT* child–Pugh score, *HCC* hepatocellular carcinoma, *LRT* locoregional treatment, *LT* liver transplant, *PEIT* percutaneous ethanol injection therapy (alcohol ablation), *MELD* model for end-stage liver disease, *RFA* radiofrequency ablation, *SD* standard deviation, *SIRT* selective internal radiotherapy, *TACE* trans-arterial chemoembolization

### Examination of top candidates in an independent HCC dataset

Several molecules that significantly differentiated recurrent from non-recurrent samples at both gene and protein level (ALDH1A1, LGALS3, LGALS3B) were selected for further investigation. In order to examine their prognostic potential, we analyzed HCC tumors from The Cancer Genome Atlas (TCGA) using KMplotter, a web-based tool that enables survival analysis across multiple cancers and datasets. These tumors represented hepatectomy specimens and not HCC explant samples. Patient samples were split into two groups according to software cut-off for ALDH1A1 (RNAseq probe#216), LGALS3 (RNAseq probe#3958), and LGALS3BP (RNAseq probe#3959). We ran multivariate survival analysis based on the high versus low expression of each of the three genes in tumors. The two groups were compared by a Kaplan–Meier survival plot, and the hazard ratio with 95% confidence intervals and log-rank p-value were calculated.

### Statistical analysis

Distributions of Western Blot ratios (LGALS3/GAPDH, ALDH1A1/GAPDH, and LGALS3/ALDH1A1) and LGALS3BP positive staining values were examined using the Shapiro-Wilks normality test. We assessed differences between groups using the independent t-test for variables following a normal distribution, and the Wilcoxon–Mann–Whitney non-parametric test for variables not following a normal distribution. Pearson correlation coefficients were calculated between Western Blot and log2-transformed LFQ protein intensity values of a particular protein, and between log2-transformed LFQ protein intensity values of different proteins of interest, using GraphPad Prism 8.0. Immunohistochemistry results were analyzed using unpaired t-test. P < 0.05 was considered significant. Data are reported as median values with interquartile range. We investigated the association of selected significant genes, proteins or clinical characteristics with the time to recurrence, at the univariate level, with the coxph function of the survival package [31] in R version 3.6. Using the same package, we plotted Kaplan–Meier survival curves, the statistical difference between the curves being calculated with survdiff function.

## Results

### Proteome analysis of recurrent and non-recurrent HCC cases

Patient characteristics are summarized in Table 1. The study included 4 HCC cases with no recurrence and 7 cases with recurrence post-LT. Tumors showed moderate differentiation and the number of lesions was comparable in the two groups (n = 4 for non-recurrent, and n = 7 for recurrent). The non-recurrent group showed a lower frequency of microvascular invasion (25% of patients) than the recurrent group (57% of patients). Overall survival differed between the two groups, with a median of 6 years in non-recurrent patients and 3 years in recurrent patients.

**Fig. 1:**
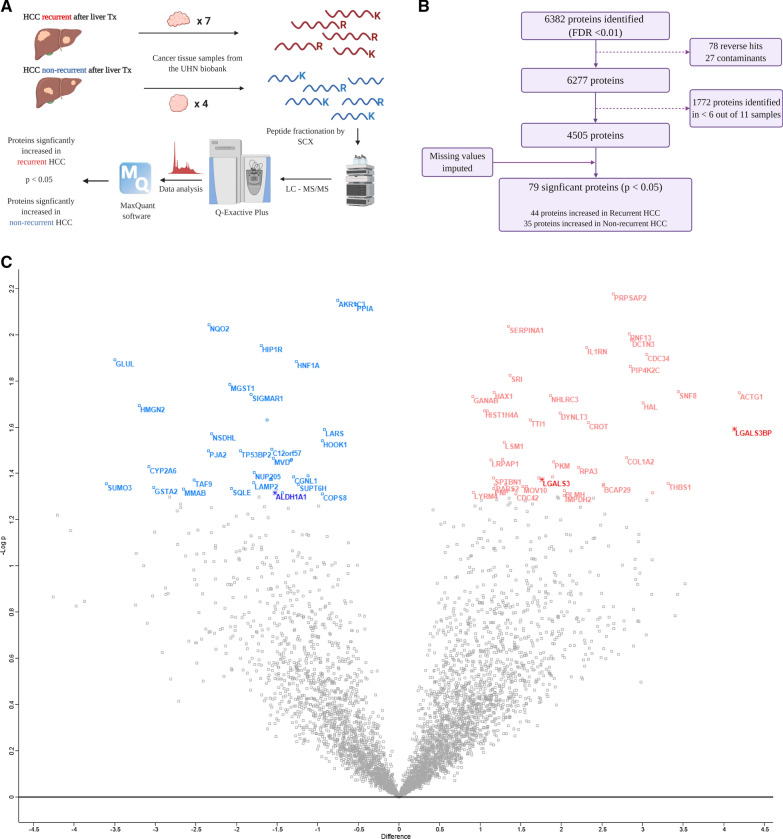
Proteomics workflow and analysis of recurrent and non-recurrent hepatocellular carcinoma (HCC) tumour explant samples obtained at the time of transplantation. **A** Schematic diagram of the proteomics workflow including the 11 HCC samples. **B** Proteomics analysis workflow to identify the most significant proteins between recurrent and non-recurrent HCC. **C** Volcano plot illustrating proteins differentially expressed (p < 0.05) in recurrent vs non-recurrent HCC tumors. Proteins in red are significantly increased in recurrent, while those in blue are significantly decreased in the recurrent cases. The three proteins bolded and marked with stars (ALDH1A1, LGALS3 and LGALS3BP) were subsequently selected for validation. Some protein names were removed for clarity, to minimize overlap. HCC, hepatocellular carcinoma; SCX, strong cationic exchange; LC, liquid chromatography; MS/MS, tandem mass spectrometry; ALDH1A1, retinal dehydrogenase 1, LGALS3, galectin-3; LGALS3BP, galectin-3-binding protein

We first analyzed the proteome of HCC explants (Fig. 1A). We identified and quantified a total of 6382 proteins (FDR < 0.01) from 11 HCC samples, using unbiased LC–MS/MS followed by label-free quantification (Fig. 1B). After removal of false-positive (reverse) hits and contaminants, 6277 proteins were further analyzed. In both study groups, the total numbers of identified proteins per sample were comparable and evenly distributed (Additional file 5: Table S1, Additional file 1: Fig. S1A, B). We next excluded from further analysis those proteins identified in < 6 samples, which resulted in 4505 proteins identified and quantified in at least 6 samples. The median number of proteins identified per sample was 4073, after filtering (Additional file 5: Table S1). Our 4505 proteins demonstrated bell-shaped distribution (Additional file 1: Fig. S1A) in each sample. We thus imputed missing protein values from a normal distribution and assumed low abundance (Additional file 1: Fig. S1B). Principal component analysis using all 4505 proteins illustrated that while most recurrent and non-recurrent samples were correctly separated using protein expression data, sample 10 was the most distinct sample (Additional file 1: Fig. S1C). We finally identified 79 proteins that were significantly differentially expressed between recurrent and non-recurrent samples (p < 0.05), with 44 proteins increased and 35 proteins decreased in recurrent samples (Additional file 1: Fig. 1C, Additional file 6: Table S2). We focused on proteins LGALS3BP (recurrent/non-recurrent fold change = 5.8, p = 0.025), member of the beta-galactoside-binding proteins that modulate cell–cell and cell–matrix interactions and LGALS3, a galectin/carbohydrate binding protein (recurrent/non-recurrent fold change = 2.3, p = 0.042), among the proteins significantly increased in recurrent samples. We also focused on ALDH1A1 (recurrent/non-recurrent = 0.2, p = 0.048), which was significantly decreased in recurrent samples. ALDH1A1 is an enzyme member of the aldehyde dehydrogenase family involved in alcohol metabolism. These proteins were selected because of their concomitant significant upregulation at the gene expression level [32–34]. Additionally, we noted that these proteins were identified confidently, with many unique peptides, and their expression levels correlated. Levels of ALDH1A1 negatively and significantly correlated with the levels of LGALS3 (Additional file 2: Fig. S2A), while the protein levels of LGALS3 and LGALS3BP showed a positive and significant correlation with each other (Additional file 2: Fig. S2B). Among the 79 proteins differentially expressed in recurrent vs non-recurrent cases, 21 pathways were significantly enriched (adj. p < 0.05) (Additional file 7: Table S3). Most proteins, including LGALS3BP and LGALS3, belonged to the “immune system” and “innate immune system” based on pathway analysis.

### Transcriptome analysis of recurrent and non-recurrent HCC cases

**Fig. 2:**
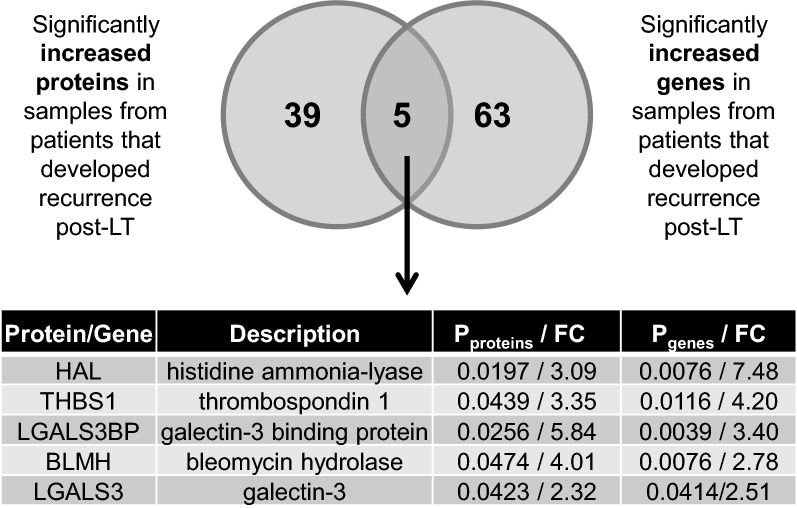
Common proteins and genes significantly increased in HCC tumour explants obtained at the time of transplant and exhibiting post-transplant recurrence. The Venn diagram illustrates the overlap between proteins and genes significantly increased in HCC tumour explant samples from patients that developed recurrence post-LT. HCC, hepatocellular carcinoma; LT, liver transplant; FC, fold change of the mean gene expression in recurrent samples, compared to non-recurrent

**Fig. 3:**
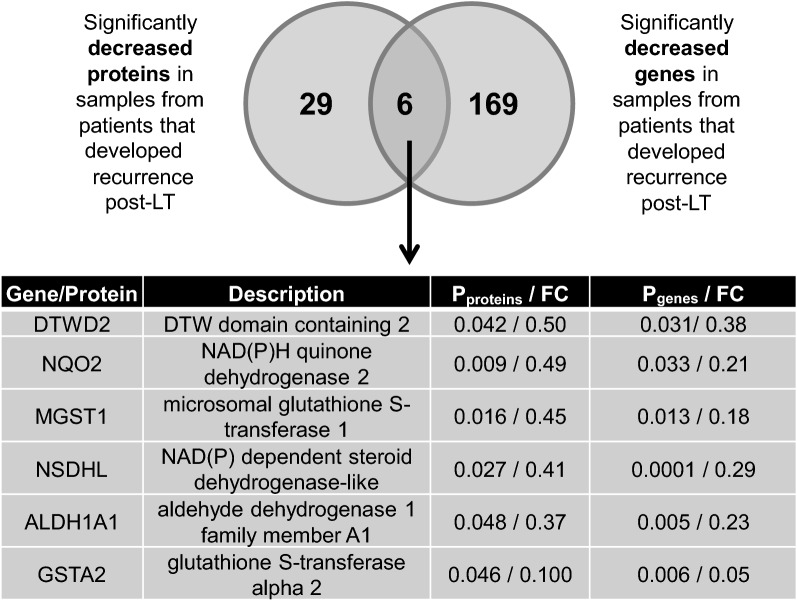
Common proteins and genes significantly decreased in HCC tumour explants obtained at the time of transplant and exhibiting post-transplant recurrence. The Venn diagram illustrates the overlap between proteins and genes significantly decreased in HCC tumour explant samples from patients that developed recurrence post-LT. HCC, hepatocellular carcinoma; LT, liver transplant; FC, fold change of the mean gene expression in recurrent samples, compared to non-recurrent

A total of 636 genes were significantly differentially expressed between the same recurrent compared to non-recurrent HCC samples. We first examined those genes that were also significantly increased at the protein level in recurrent HCC. Sixty-eight upregulated genes and 44 upregulated proteins were identified in HCC explants associated with post-transplant recurrence, with five molecules in alignment with our proteome findings (Fig. 2). The common genes/proteins increased in recurrent HCC were significantly associated with cancer signaling pathways including PI3K-Akt signaling pathway (p = 0.002) and Ras signaling pathway (p = 0.010) (Additional file 8: Table S4), as well as TGF-β signaling pathway (p = 0.0099) and extracellular matrix (ECM) receptor interaction (p = 0.0099). Metabolic pathways (p = 2.10E-06), Steroid biosynthesis (p = 3.70E-05), Fatty acid degradation (p = 0.0003) and Glycolysis/Gluconeogenesis (p = 0.0008) were the most significantly enriched, as determined by the common elements (n = 6) of the genes (n = 175) and proteins (n = 35) significantly decreased in recurrent HCC (Additional file 8: Table S5, Fig. 3).

### ALDH1A1, LGALS3, and LGALS3BP are predictors of HCC recurrence

**Fig. 4:**
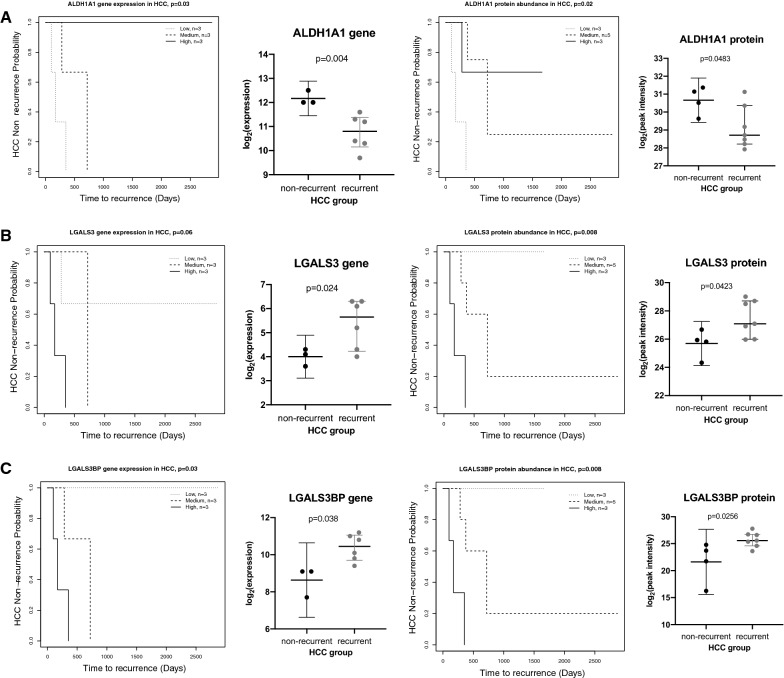
Kaplan–Meier and dot plots for: **A** ALDH1A1 gene and protein; **B** LGALS3 gene and protein; **C** LGALS3BP gene and protein in HCC patients. The levels of the gene/protein were grouped by interquartile ranges. For ALDH1A1 gene: low level ≤ 10.40; medium level [10.40–12.00]; high level ≥ 12.00. For ALDH1A1 protein: low level ≤ 28.59; medium level [28.59–30.82]; high level ≥ 30.82. For LGALS3 gene: low level ≤ 4.10; medium level [4.10–6.10]; high level ≥ 6.10. For LGALS3 protein: low level ≤ 25.88; medium level [25.88–27.80]; high level ≥ 27.80. For LGALS3BP gene: low level ≤ 9.10; medium level [9.10–10.80]; high level ≥ 10.80. For LGALS3BP protein: low level ≤ 22.69; medium level [22.69–26.09]; high level ≥ 26.09. LGALS3, galectin-3; LGALS3BP, galectin-3-binding protein

Molecules that significantly differentiated recurrent and non-recurrent samples at both gene and protein level, together with the selected clinical variables (Table 2) were investigated at univariate level. High levels of the ALDH1A1 gene and protein were negatively associated with the HCC recurrence. On the other hand, both LGALS3BP and LGALS3 gene and protein levels were positively associated with HCC recurrence (Fig. 4). Except for age, no other clinical variable, including the RETREAT score that was developed for the HCC within Milan patients, was predictive for HCC recurrence. We noted that the RETREAT score was not significantly different between patients with recurrent HCC vs non-recurrent (p-value = 0.068). In addition, we plotted survival curves using the mean value as cut-off (Additional file 3: Fig. S3). The results show that ALDH1A1 protein, LGALS3 protein and LGALS3BP gene were still significantly associated with HCC non-recurrence. Due to small sample size, we did not perform multivariable analysis.

**Table 2:** Univariate analysis of the top proteins and genes differentially expressed in HCC explants and key clinical characteristics, as predictors of HCC recurrence post-transplant

Variable	HR (95% CI)	p-value
**DTWD2 (protein)**	**0.15 (0.032–0.69)**	**0.014**
**LGALS3 (protein)**	**2.6 (1.2–5.5)**	**0.015**
**ALDH1A1 (gene)**	**0.084 (0.01–0.68)**	**0.02**
**NSDHL (protein)**	**0.63 (0.42–0.94)**	**0.026**
**BLMH (protein)**	**1.6 (1–2.5)**	**0.03**
**ALDH1A1 (protein)**	**0.39 (0.16–0.91)**	**0.03**
**Age**	**0.85 (0.74–0.98)**	**0.03**
**LGALS3BP (gene)**	**7.1 (1.2–43)**	**0.032**
**LGALS3BP (protein)**	**2.6 (1.1–6.1)**	**0.036**
**LGALS3 (gene)**	**2.9 (1–8.3)**	**0.05**
**HAL (protein)**	**1.4 (1–2.1)**	**0.05**
RETREAT score	2 (0.94–4.3)	0.074
THBS1 (protein)	1.5 (0.96–2.4)	0.077
DTWD2 (gene)	0.17 (0.023–1.2)	0.08
GSTA2 (gene)	0.72 (0.49–1)	0.081
MGST1 (protein)	0.67 (0.42–1.1)	0.094
HAL (gene)	1.9 (0.88–4.2)	0.1
BLMH (gene)	2.3 (0.85–6)	0.1
NQO2 (protein)	0.57 (0.29–1.1)	0.11
NQO2 (gene)	0.44 (0.15–1.3)	0.14
NSDHL (gene)	0.045 (0.00051–3.9)	0.17
THBS1 (gene)	1.7 (0.79–3.5)	0.18
GSTA2 (protein)	0.83 (0.62–1.1)	0.19
AFP	1 (1–1)	0.23
MGST1 (gene)	0.55 (0.2–1.5)	0.24
microvascular invasion	2.2 (0.45–10)	0.33
No. of tumors	1.1 (0.93–1.2)	0.42

The significant molecular and clinical variables are listed in bold

*HR* hazard ratio, *CI* confidence interval

### Verification and validation of ALDH1A1, LGALS3 and LGALS3BP changes in recurrent and non-recurrent HCC

**Fig. 5:**
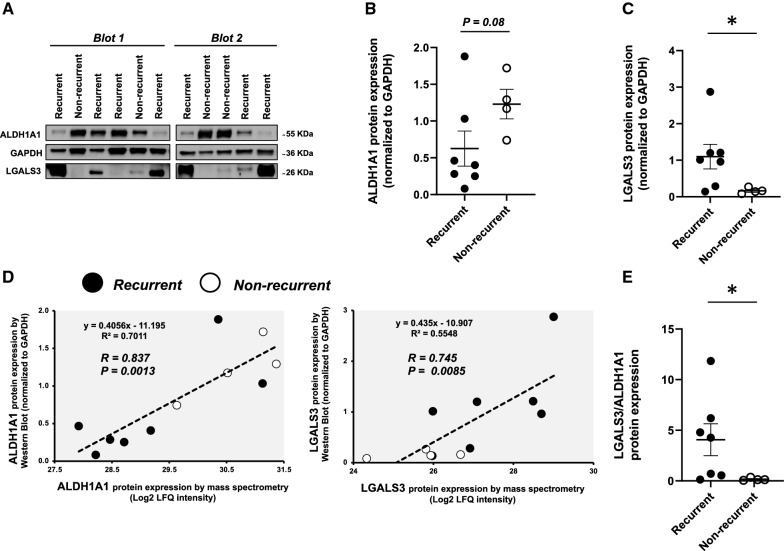
Verification of ALDH1A1 and LGALS3 protein expression changes in HCC tumour explants. **A** Shows the immunoblot bands of ALDH1A1, LGALS3, and GAPDH in recurrent (n = 7) and non-recurrent (n = 4) HCC patients. Cropped images of the blots were used in order to improve the clarity and conciseness of the presentation. The protein expression of ALDH1A1 (**B**) and LGALS3 (**C**) were measured by densitometry and normalized to GAPDH. The Pearson correlations between Western Blot and mass spectrometry log2-transformed LFQ protein intensity values of ALDH1A1 and LGALS3 were evaluated (**D**). The Western blot LGALS3/ALDH1A1 ratio was also calculated (**E**). *P < 0.05. Data are reported as mean ± standard error. LFQ, label-free quantification; LGALS3, galectin-3; ALDH1A1, retinal dehydrogenase 1. LGALS3, galectin-3; LGALS3BP, galectin-3-binding protein; ALDH1A1, retinal dehydrogenase 1

We employed Western Blot to confirm the observed changes in the protein expression of ALDH1A1, LGALS3 and LGALS3BP. Unfortunately, the antibody against LGALS3BP did not generate a single clear band, forcing us to focus on the first two proteins. In keeping with our proteomics findings, ALDH1A1 protein expression was numerically increased in non-recurrent samples (P = 0.085; Fig. 5A, B), while LGALS3 expression was significantly increased in recurrent samples (P = 0.027; Fig. 5A, C). Reassuringly, there was a strong, significant, and direct correlation between protein intensity levels of ALDH1A1 and LGALS3 measured by mass spectrometry, and their corresponding protein expression by Western Blot (R = 0.837, P = 0.001 for ALDH1A1; R = 0.745, P = 0.008 for LGALS3; Fig. 5D). Moreover, mass spectrometry intensity levels of ALDH1A1 negatively and significantly correlated with mass spectrometry levels of LGALS3 (R = − 0.838, P = 0.001; Additional file 2: Fig. S2A). Since ALDH1A1 and LGALS3 were differentially expressed in opposite directions between recurrent and non-recurrent samples, we evaluated the LGALS3/ALDH1A1 ratio. Importantly, we observed a > 20-fold significant increase in the LGALS3/ALDH1A1 Western Blot ratio in recurrent, as compared to non-recurrent samples (P = 0.012; Fig. 5E).

**Fig. 6:**
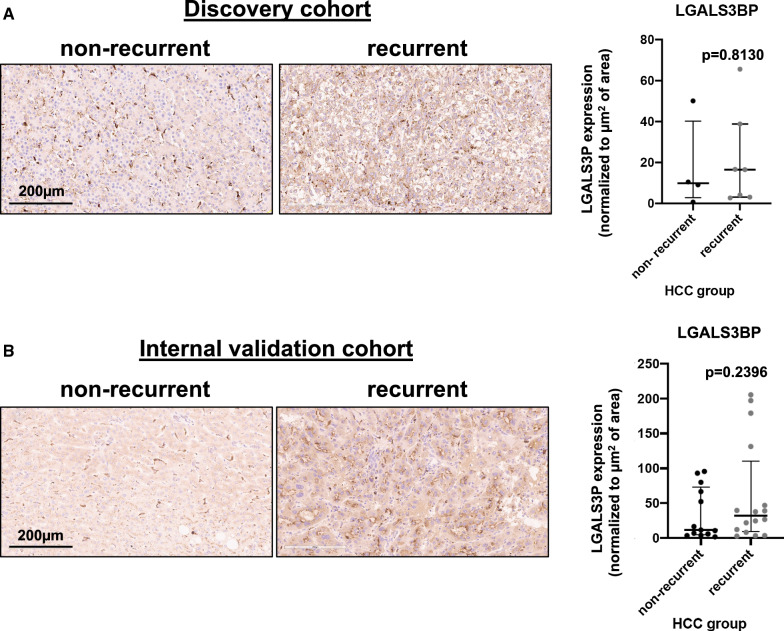
Validation of LGALS3B protein expression changes in HCC tumour explants. Increased protein expression of LGALS3BP in HCC tumour explant samples from patients that developed recurrence post-LT (n = 7), compared to non-recurrent cases (n = 4), were orthogonally verified by immunohistochemistry in the discovery cohort (**A**). The same trend was observed in an independent, internal validation cohort of 16 recurrent and 13 non-recurrent HCC cases. Magnification: 20x. Scale bar: 200 μm. HCC, hepatocellular carcinoma; LGALS3BP, galectin-3-binding protein

We next employed immunohistochemistry to confirm the observed changes in the protein expression of LGALS3BP. In concordance with our observations at the proteome and transcriptome level, LGALS3BP positive staining was numerically increased in recurrent HCC tumor explants, compared to non-recurrent, when examining the 11 samples from our discovery cohort (Fig. 6A). Reassuringly, increased LGALS3BP staining was also observed among recurrent cases in an independent, internal cohort of 29 LT recipients with HCC beyond Milan criteria (Fig. 6B).

### Examination of top candidates in an independent HCC dataset

The impact of ALDH1A1, LGALS3 and LGALS3BP gene expression on overall survival was analyzed using the TCGA HCC dataset of 364 patients with HCC. ALDH1A1 was significantly predictive of overall survival in HCC patients, with a hazard ratio of 0.66 (95%CI 0.46–0.95, logrank p = 0.024) being associated with high expression level (Additional file 4: Fig. S4A). In contrast, increased LGALS3 expression (Additional file 4: Fig. S4B) was associated with an increased likelihood of death, with a hazard ratio of 1.59 (95%CI 1.09–2.3, logrank p = 0.014). Similarly, high expression of LGALS3BP (Additional file 4: Fig. S4C) was associated with increased likelihood of death, with a hazard ratio of 1.36 (95%CI 0.94–1.97), although below significance threshold (logrank p = 0.1)**.** These findings in the independent dataset suggest that the expression of ALDH1A1, LGALS3 and LGALS3BP may be indicative of the aggressiveness of HCC, and thus its inherent propensity to recur post-transplant.

## Discussion

We have delineated a set of proteins/genes on liver explant predictive of recurrence post-transplant, in patients transplanted for HCC beyond Milan. We found that expression levels of 3 specific proteins and their transcripts (ALDH1A1, LGALS3 and LGALS3BP) distinguish those patients with recurrence post-transplant from those who did not recur. These findings were orthogonally validated in an independent cohort of recipients transplanted for HBV-HCC beyond Milan. These analytes represent a potential prognostic immunohistochemical signature in HCC beyond Milan criteria.

Our combined proteomic/transcriptomic signature adds to the current knowledge on predicting recurrence post-LT in HCC beyond Milan criteria. Current predictors of HCC recurrence after LT have been limited to tumor burden on the explant, presence of vascular invasion, and elevated AFP, together comprising scores such as the RETREAT score, used to determine the frequency of surveillance for post-LT recurrence [35]. However, this cannot be used in pre-transplant decision making, as microvascular invasion is only confirmed by post-transplant explant histological assessment. This denotes the limitations of the current image-based only selection algorithm of HCC for LT. Additionally, the ability to predict recurrence is critical for a few reasons: first, those beyond Milan HCC with favourable molecular signatures could be considered for LT. Secondly, the risk-to-benefit ratio might not be in favour of transplant in those with poor molecular features. Those with a higher risk of recurrence would need to be considered for post-transplant surveillance.

We found that high expression of ALDH1A1 protein and transcript level in the primary HCC tumor was associated with lower rates of recurrence post-LT. The expression of ALDH1A1 has been reported as a favorable prognostic factor in HCC, being significantly associated in previous literature with low serum levels of alpha–fetoprotein, well-differentiated pathology and a favorable clinical outcome [36–38]. Similarly, ALDH1A1 had significantly downregulated phosphorylation in HCC compared to adjacent tissue [11]. Consistent with our results, in a gene ontology annotation study of the ALDH1 family [39], HBV–related HCC patients who showed high ALDH1L1 gene expression had superior clinical outcome, with a 57–month recurrence–free survival. High ALDH1B1 expression was protective for HCCs with multiple nodules and high serum alpha–fetoprotein level [39]. Guo et al. [40] have previously reported that long noncoding RNA MACC1-AS1 promoted the stemness of HCC cells by antagonizing ALDH1 [39]. Our study in the LT context is thus in keeping with previous literature on ALDH1A1 in non-transplant HCC.

ALDH1A1 catalyzes the oxidation of the retinol metabolite retinaldehyde to retinoic acid [33]. Thus, lower ALDH1A1 expression in our recurrent cases could represent reduced production of retinoic acid. In concordance, experimental evidence shows that retinoid activity is reduced in HCC cell lines. Moreover, patients with HCC display decreased hepatic retinoid stores and altered retinoid signaling [41]. Intriguingly, retinoic acid treatment inhibited LGALS3 expression in carcinoma cells [42], suggesting that reduced levels of retinoic acid could promote LGALS3 upregulation. In turn, increased levels of LGALS3 have been associated with a higher ability of the tumors to neutralize the immune system [32]. ALDH1A1 and LGALS3 were differentially expressed in opposite directions between recurrent and non-recurrent samples, and their expression strongly and negatively correlated with each other. In addition, we demonstrated a significant increase in the LGALS3/ALDH1A1 ratio in recurrent samples, as compared to non-recurrent samples. This finding may suggest that ALDH1A1 and LGALS3 are functionally related and contribute to HCC invasiveness, particularly in tumours with high LGALS3 relative to ALDH1A1 expression.

LGALS3 expression levels were directly associated with a higher risk of HCC recurrence post-transplant in our cohort. LGALS3 was reported to be critical for Ras signaling and thus supports mitogen activated protein kinase (MAPK) and AKT cascades. Increased levels of LGALS3 have been associated with a higher ability of the tumors to neutralize the immune system [43]. Along the same lines, LGALS3 protein expression was increased in HCC tumours, compared to adjacent tissue [11]. The same study demonstrated that both LGALS3 and LGALS3BP were significantly increased in more aggressive HCC tumours. Our survival investigation on an external HCC dataset from TCGA showed that, despite these samples representing a clinical context different from our own, the results were supportive, by revealing worse prognosis of tumors expressing high LGALS3 and LGALS3BP and low ALDH1A1. LGALS3BP interacts with LGALS3 and was found to be upregulated and highly correlated with LGALS3 in our study. Prior studies indicated that LGALS3BP can be used along with alpha-fetoprotein to improve the screening sensitivity for HCC [43]. LGALS3BP, together with CD5 antigen like (CD5L) and immunoglobulin J chain (IGJ) constituted a triple-marker panel predictive of poor HCC survival and rapid progression after sorafenib treatment [44]. LGALS3 and LGALS3BP are thus candidate proteins for identifying HCC tumours with increased likelihood for recurrence post-LT, which might represent potential therapeutic targets to diminish HCC recurrence post-LT [45].

Other studies have examined transcriptional markers of HCC recurrence post-LT. Miltiadous et al. [46] conducted a pivotal study examining gene expression profiling of HCC beyond the Milan Criteria and identified the progenitor phenotype (determined by either CK‐19 expression or the Hoshida S2 signature) as predictive of post-LT recurrence. The S2 HCC subclass was linked to high levels of alpha–fetoprotein expression, activation of mTOR and IGF signaling, even at the early stages of the disease. This study certainly pinpointed potential gene signatures predictive of recurrence. Importantly, our study has extended this approach by generating a combined transcriptomic and proteomic signature predictive of HCC recurrence post-LT, which permits cost-effective immunohistochemical evaluation of a proteomic signature.

Our study had limitations. First, our sample size was limited, and it was not possible to adjust for significant clinical and pathological covariates such as tumor burden on explant, presence of microvascular invasion and AFP at time of transplant. Nonetheless, we included only samples from Hepatitis B-induced HCC, the most common reason for HCC liver transplant beyond Milan criteria, in order to minimize heterogeneity. This is a pilot study and additional studies with larger sample sizes and etiologies beyond hepatitis B will be needed to validate our results. Additionally, with long pre-LT waiting times before transplantation and bridging therapy, the stability of tumor gene/protein expression profiles over time must be considered, should predictive signatures be incorporated into pre-LT patient selection. Despite these shortcomings, our observations provide an initial comprehensive and combined proteomic and transcriptomic profile of HCC explants for patients identified pre-LT beyond Milan criteria. Integration of high-throughput omics profiles is particularly powerful to decipher the key molecules involved in carcinogenesis [47–50]. Because expression profiling could be performed on liver needle biopsies in a pretransplant setting [51], the positivity of the proposed signatures might also assist in the decision making for selecting patients for transplantation.

In conclusion, we have derived an HCC explant protein signature comprised of ALDH1A1, LGALS3 and LGALS3BP to predict risk of HCC recurrence post-transplant. This short list of proteins was identified using both high-throughput proteomics and transcriptomics, and validated using immunohistochemistry and immunoblotting on HCC explant specimens. Staining of explant specimens for these 3 proteins could provide further guidance regarding screening protocols for patients transplanted for HCC beyond Milan. These findings support the possibility that molecular analysis of HCC performed prior to LT may enable expansion of the current imaging-based selection criteria to include patients beyond Milan Criteria with favorable molecular profiles, though this will require further investigation.

## Supplementary Information

**Additional file 1: Figure S1.** Distribution of protein intensity values and principal component analysis of the HCC samples. Each histogram in panel A represents the distribution of the original log2 transformed LFQ intensity values among the proteins quantified in each of the 11 studied HCC tumor explant samples. Blue bars represent the count of intensity values determined by mass spectrometry. In panel B, red bars represent the distribution of the imputed intensity values. To evaluate the similarity across the proteomes of the studied samples, the distribution of variances of the log2 transformed LFQ intensity values of all quantified proteins were examined by principal component analysis using Perseus software (C). The tumor explant samples from LT patients with recurrent HCC are depicted in red, while the non-recurrent cases are represented in blue. LFQ, label-free quantification; LT, liver transplant; HCC, hepatocellular carcinoma.

**Additional file 2: Figure S2.** Correlation analysis between the immunoblotting and mass spectrometry-based protein levels of key proteins identified in this study. The Pearson correlation coefficients (R), as well as the significance of the correlation (P), were calculated for the following comparisons: LGALS3 vs. LGALS3BP protein intensity (A, n = 10), LGALS3 vs. ALDH1A1 protein intensity (B, n = 11), and ALDH1A1 vs. LGALS3BP protein intensity (C, n = 10). P < 0.05 was considered statistically significant. LFQ, label-free quantification; ALDH1A1, retinal dehydrogenase 1, LGALS3, galectin-3; LGALS3BP, galectin-3-binding protein.

**Additional file 3: Figure S3.** Kaplan–Meier survival plots for: (A) ALDH1A1 gene and protein; (B) LGALS3 gene and protein; (C) LGALS3BP gene and protein in HCC patients. The levels of the gene/protein were grouped by mean. For ALDH1A1 gene: low level < 11.22; high level ≥ 11.22. For ALDH1A1 protein: low level < 29.67; high level ≥ 29.67. For LGALS3 gene: low level < 4.91; high level ≥ 4.91. For LGALS3 protein: low level < 26.60; high level ≥ 26.60. For LGALS3BP gene: low level < 9.80; high level ≥ 9.80. For LGALS3BP protein: low level < 23.48; high level ≥ 23.48.

**Additional file 4: Figure S4.** Kaplan–Meier survival curves based on gene expression of: (A) ALDH1A1, (B) LGALS3 and (C) LGALS3BP in the TCGA HCC dataset**.** HR, hazard ratio; ALDH1A1, retinal dehydrogenase 1; LGALS3, galectin-3; LGALS3BP, galectin-3-binding protein.

**Additional file 5: Table S1.** Total number of proteins identified in each HCC explant sample before and after filtering.

**Additional file 6: Table S2.** Proteins significantly differentially expressed in HCC samples that go on to develop HCC recurrence compared to those that do not recur. NR, non-recurrence; R, recurrence.

**Additional file 7: Table S3.** Significantly enriched pathways among proteins differentially expressed between HCC samples developing recurrence compared to non-recurrent ones. Pathways are determined using pathDIP, and those with adjusted p-value < 0.05 by Benjamini–Hochberg are included.

**Additional file 8: Table S4.** List of pathways associated with gene/proteins significantly increased in HCC explant samples that exhibited post-transplant recurrence. **Table S5.** List of pathways associated with genes/proteins significantly decreased in HCC explant samples that exhibited post-transplant recurrence. **Table S6.** Clinical characteristics of the 29 patients transplanted for HCC beyond Milan criteria with versus without recurrence post-transplant. Median values are reported, and the 95% confidence intervals are displayed in the brackets.

## Data Availability

The transcriptomic data have been deposited to Gene Expression Ominibus (GEO) repository with the dataset identifier GSE164368. The mass spectrometry proteomics data have been deposited to the ProteomeXchange Consortium via the PRIDE [24] partner repository with the dataset identifier PXD022881 (Reviewer account details: Username: reviewer_pxd022881@ebi.ac.uk; Password: 2GmXJTJo).
