# Supplementary figures and images for: Combined proteomic/transcriptomic signature of recurrence post-liver transplantation for hepatocellular carcinoma beyond Milan
Source: Clin Proteomics. 2021 Nov 18;18:27. doi: 10.1186/s12014-021-09333-x (PMC8600773; doi:10.1186/s12014-021-09333-x)

FIGURE S1

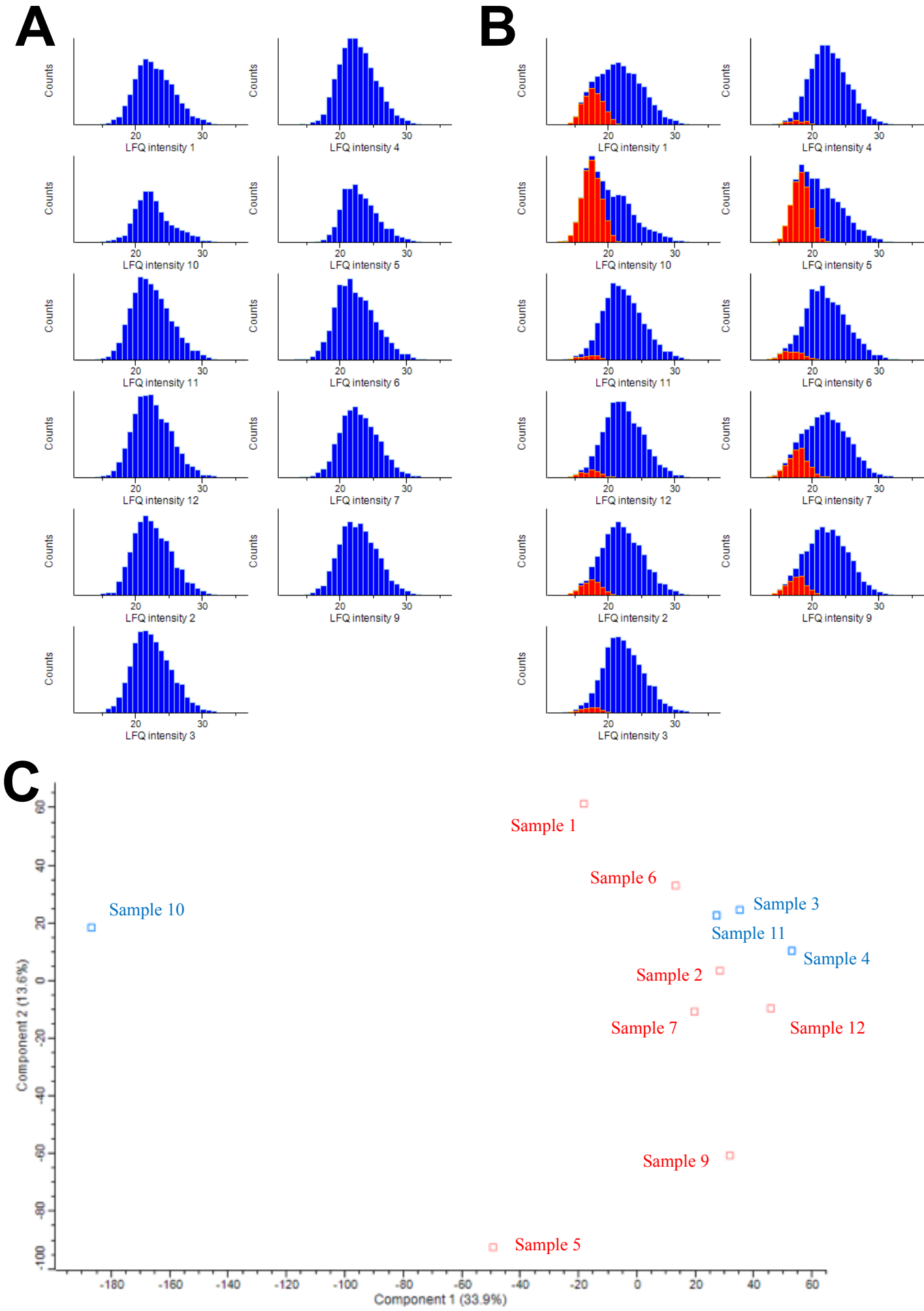

Supplement: Supplementary file 1 — Additional file 1: Figure S1. Distribution of protein intensity values and principal component analysis of the HCC samples. Each histogram in panel A represents the distribution of the original log2 transformed LFQ intensity values among the proteins quantified in each of the 11 studied HCC tumor explant samples. Blue bars represent the count of intensity values determined by mass spectrometry. In panel B, red bars represent the distribution of the imputed intensity values. To evaluate the similarity across the proteomes of the studied samples, the distribution of variances of the log2 transformed LFQ intensity values of all quantified proteins were examined by principal component analysis using Perseus software (C). The tumor explant samples from LT patients with recurrent HCC are depicted in red, while the non-recurrent cases are represented in blue. LFQ, label-free quantification; LT, liver transplant; HCC, hepatocellular carcinoma. [file 12014_2021_9333_MOESM1_ESM.pdf]

● *Recurrent* ○ *Non-recurrent*

**A**

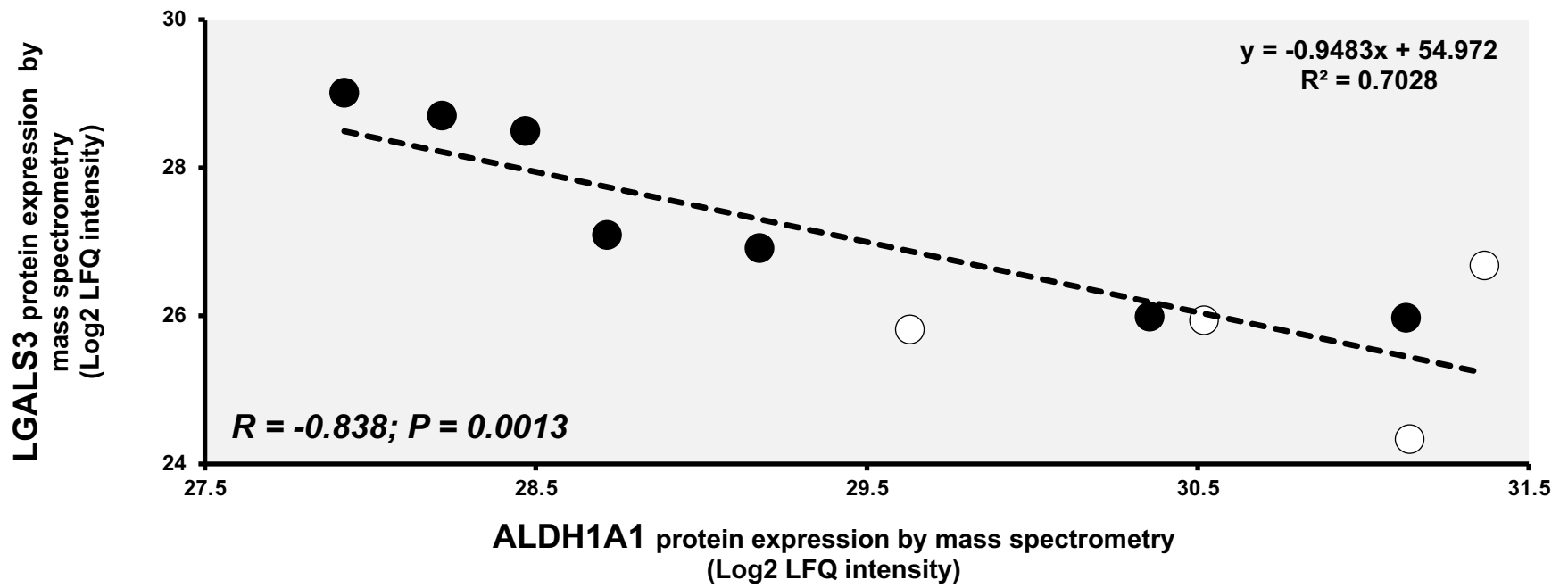

**B**

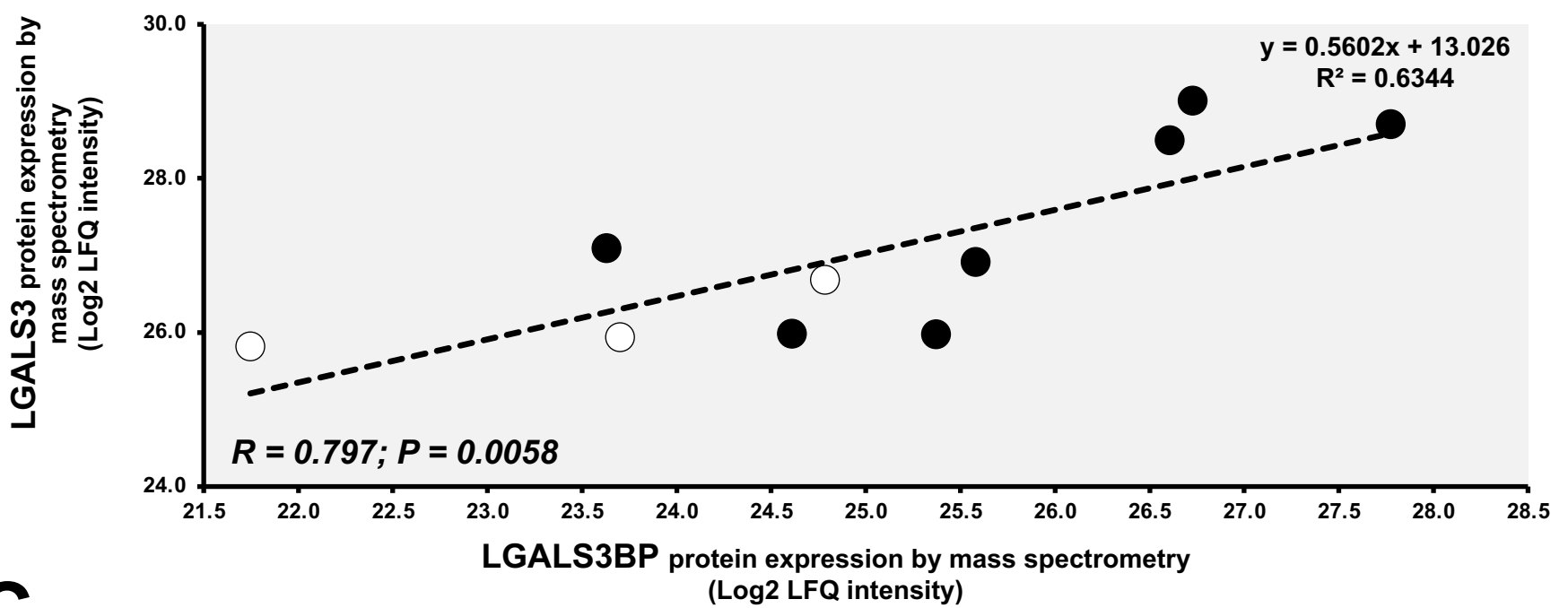

**C**

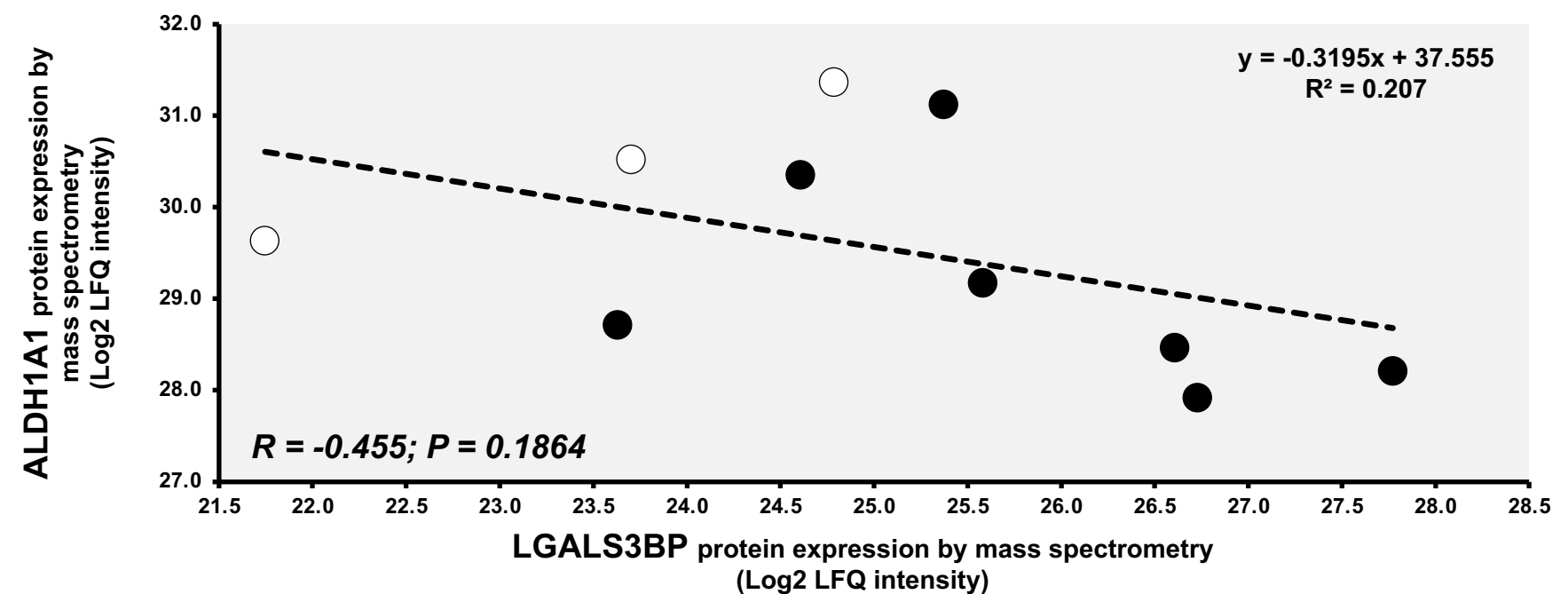

Supplement: Supplementary file 2 — Additional file 2: Figure S2. Correlation analysis between the immunoblotting and mass spectrometry-based protein levels of key proteins identified in this study. The Pearson correlation coefficients (R), as well as the significance of the correlation (P), were calculated for the following comparisons: LGALS3 vs. LGALS3BP protein intensity (A, n = 10), LGALS3 vs. ALDH1A1 protein intensity (B, n = 11), and ALDH1A1 vs. LGALS3BP protein intensity (C, n = 10). P < 0.05 was considered statistically significant. LFQ, label-free quantification; ALDH1A1, retinal dehydrogenase 1, LGALS3, galectin-3; LGALS3BP, galectin-3-binding protein. [file 12014_2021_9333_MOESM2_ESM.pdf]

**A****ALDH1A1 (216)**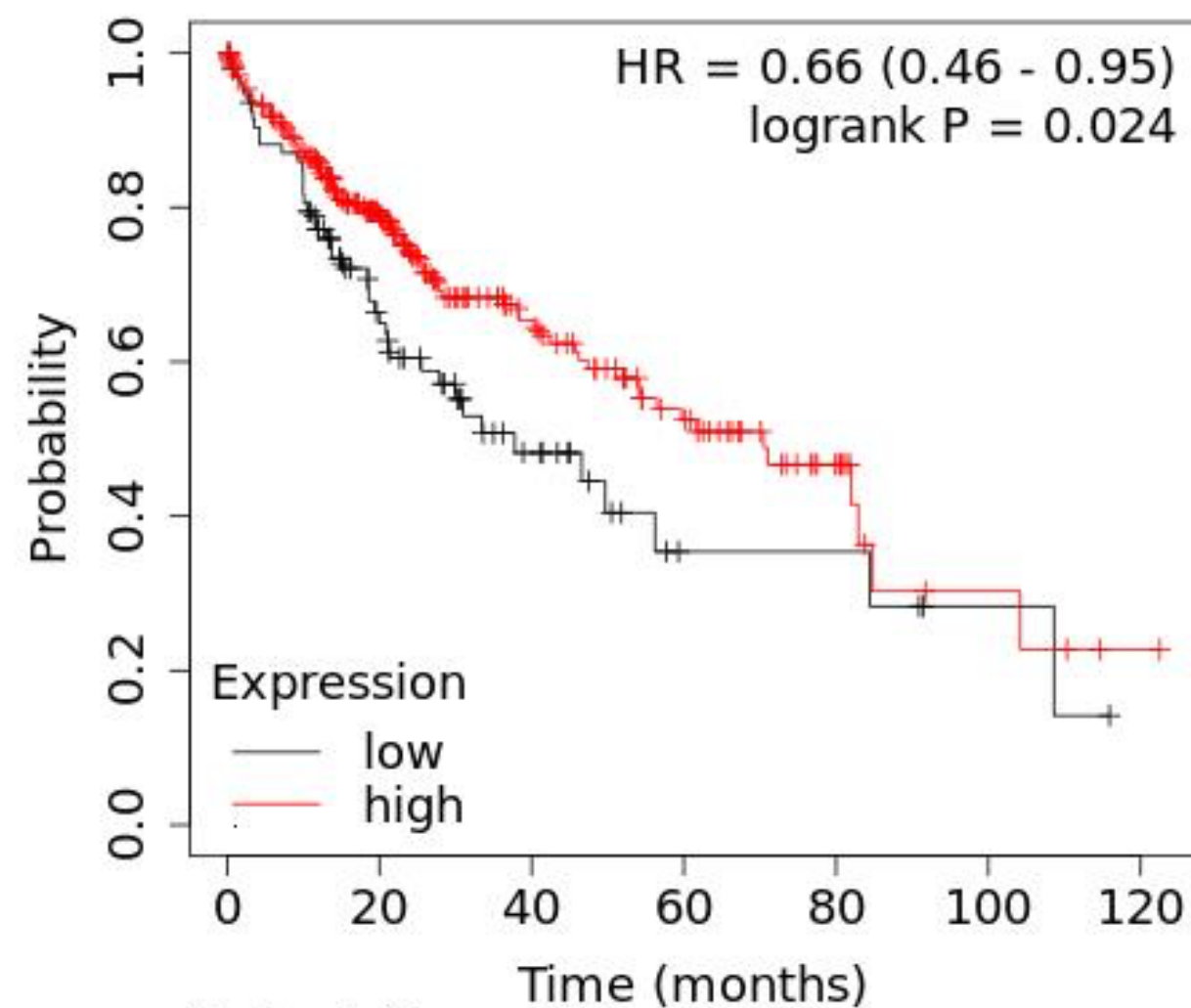**B****LGALS3 (3958)**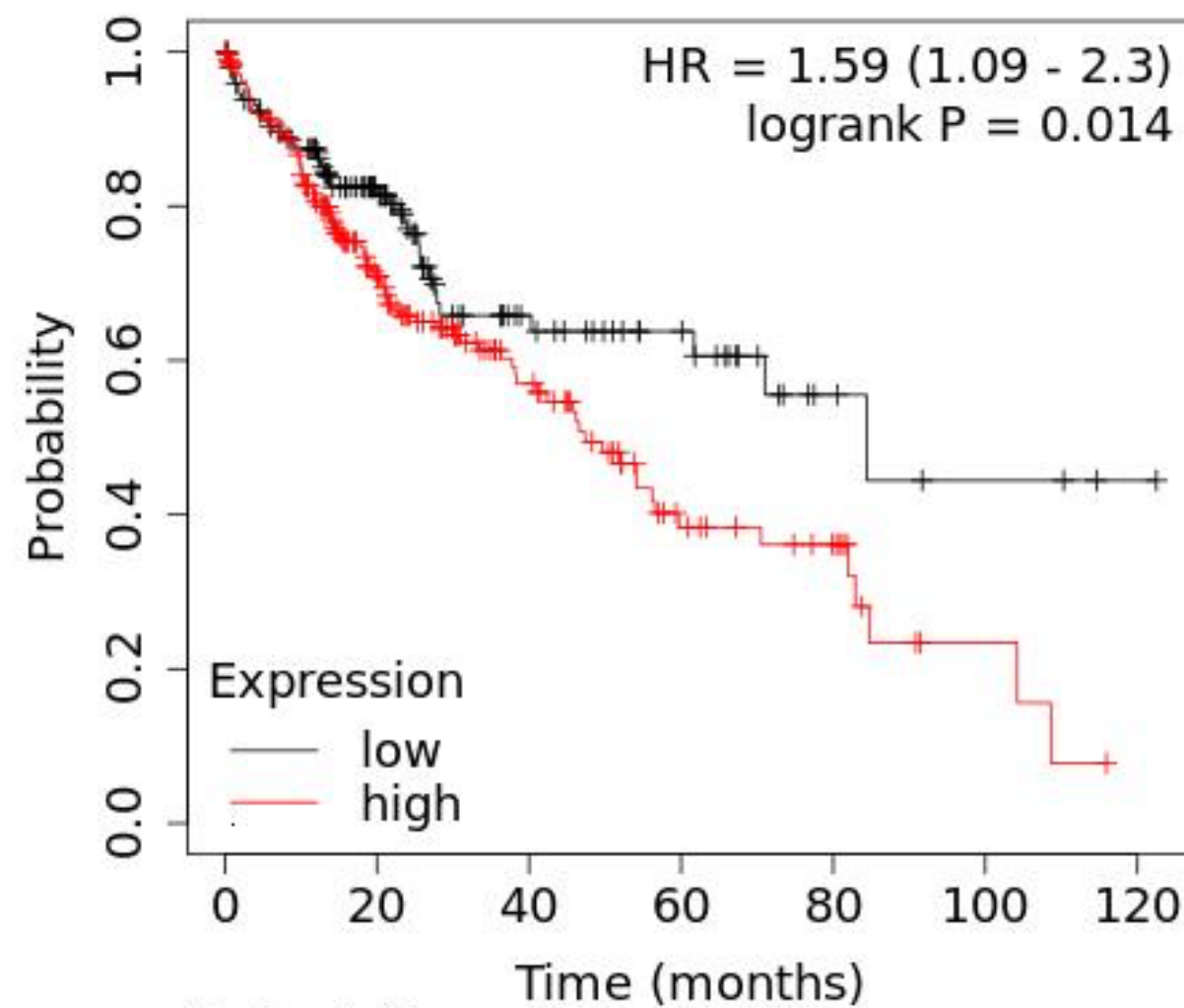**C****LGALS3BP (3959)**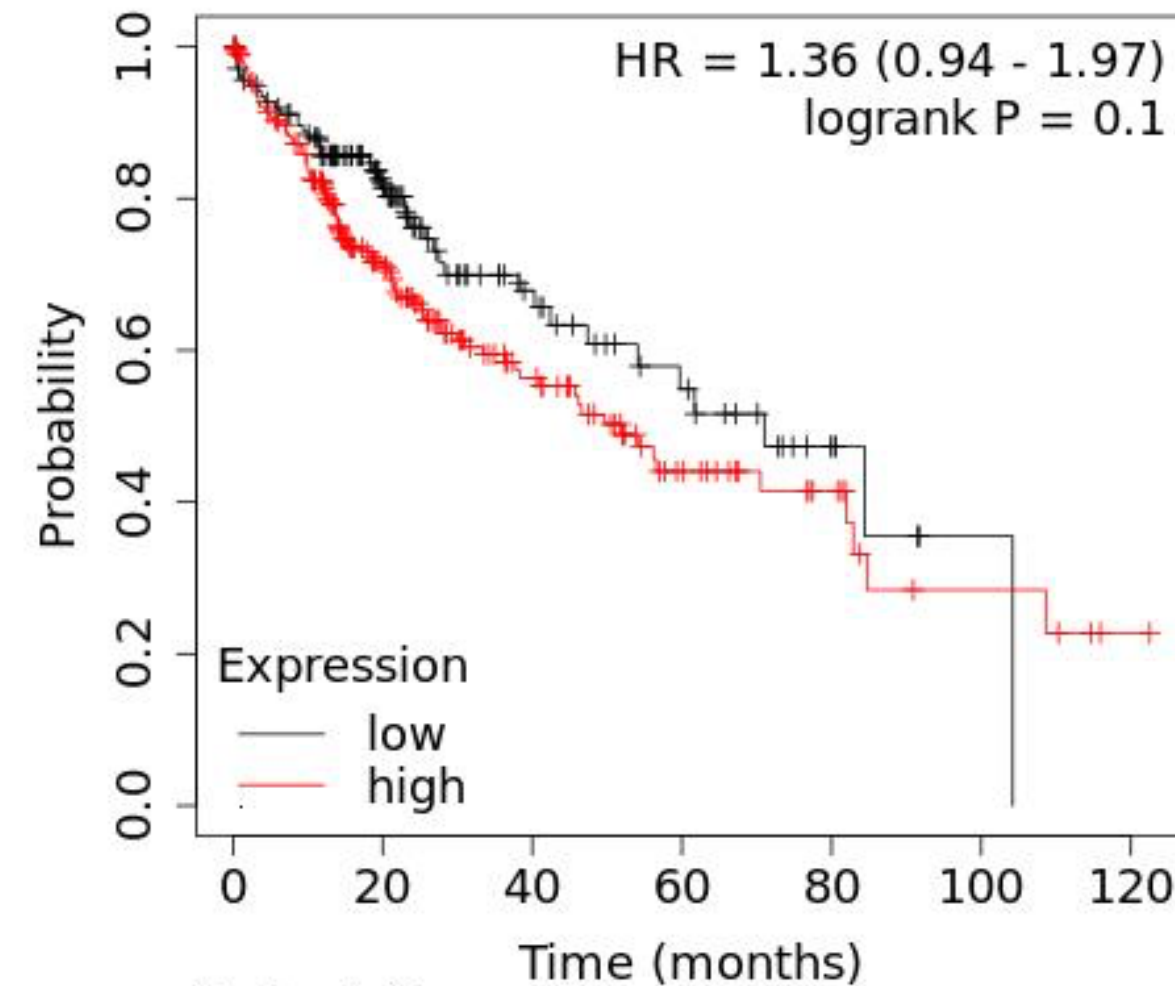

Supplement: Supplementary file 4 — Additional file 4: Figure S4. Kaplan–Meier survival curves based on gene expression of: (A) ALDH1A1, (B) LGALS3 and (C) LGALS3BP in the TCGA HCC dataset. HR, hazard ratio; ALDH1A1, retinal dehydrogenase 1; LGALS3, galectin-3; LGALS3BP, galectin-3-binding protein. [file 12014_2021_9333_MOESM4_ESM.pdf]
