# Supplementary material for: Combined proteomic/transcriptomic signature of recurrence post-liver transplantation for hepatocellular carcinoma beyond Milan
Source: Clin Proteomics. 2021 Nov 18;18:27. doi: 10.1186/s12014-021-09333-x (PMC8600773; doi:10.1186/s12014-021-09333-x)
Supplement: Supplementary file 3 — Additional file 3: Figure S3. Kaplan–Meier survival plots for: (A) ALDH1A1 gene and protein; (B) LGALS3 gene and protein; (C) LGALS3BP gene and protein in HCC patients. The levels of the gene/protein were grouped by mean. For ALDH1A1 gene: low level < 11.22; high level ≥ 11.22. For ALDH1A1 protein: low level < 29.67; high level ≥ 29.67. For LGALS3 gene: low level < 4.91; high level ≥ 4.91. For LGALS3 protein: low level < 26.60; high level ≥ 26.60. For LGALS3BP gene: low level < 9.80; high level ≥ 9.80. For LGALS3BP protein: low level < 23.48; high level ≥ 23.48. [file 12014_2021_9333_MOESM3_ESM.pdf]

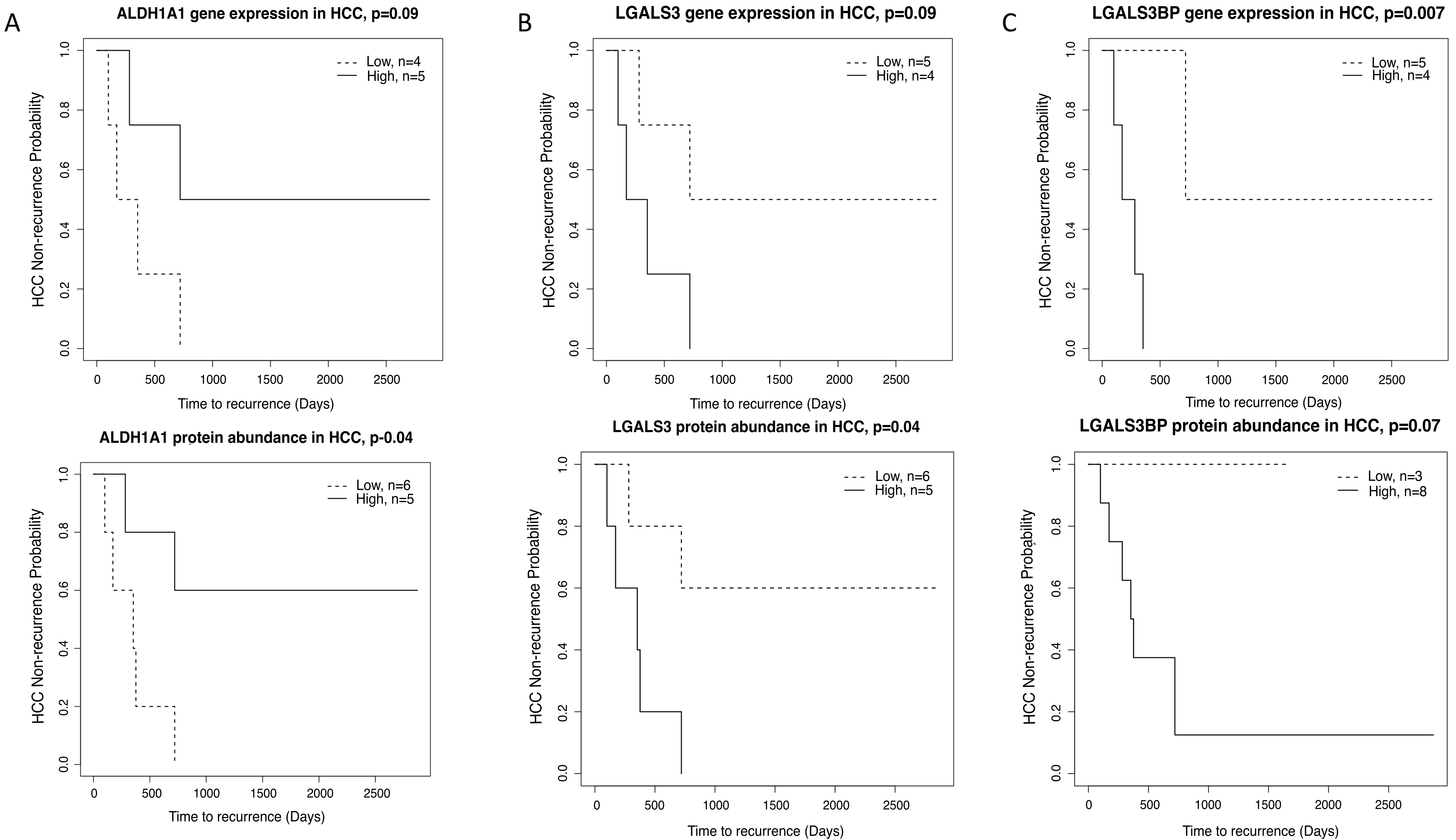
A
ALDH1A1 gene expression in HCC, p=0.09
Low, n=4
High, n=5
HCC Non-recurrence Probability
Time to recurrence (Days)
B
LGALS3 gene expression in HCC, p=0.09
Low, n=5
High, n=4
HCC Non-recurrence Probability
Time to recurrence (Days)
C
LGALS3BP gene expression in HCC, p=0.007
Low, n=5
High, n=4
HCC Non-recurrence Probability
Time to recurrence (Days)
ALDH1A1 protein abundance in HCC, p-0.04
Low, n=6
High, n=5
HCC Non-recurrence Probability
Time to recurrence (Days)
LGALS3 protein abundance in HCC, p=0.04
Low, n=6
High, n=5
HCC Non-recurrence Probability
Time to recurrence (Days)
LGALS3BP protein abundance in HCC, p=0.07
Low, n=3
High, n=8
HCC Non-recurrence Probability
Time to recurrence (Days)
