# Supplementary material for: Combined proteomic/transcriptomic signature of recurrence post-liver transplantation for hepatocellular carcinoma beyond Milan
Source: Clin Proteomics. 2021 Nov 18;18:27. doi: 10.1186/s12014-021-09333-x (PMC8600773; doi:10.1186/s12014-021-09333-x)
Supplement: Supplementary file 8 — Additional file 8: Table S4. List of pathways associated with gene/proteins significantly increased in HCC explant samples that exhibited post-transplant recurrence. Table S5. List of pathways associated with genes/proteins significantly decreased in HCC explant samples that exhibited post-transplant recurrence. Table S6. Clinical characteristics of the 29 patients transplanted for HCC beyond Milan criteria with versus without recurrence post-transplant. Median values are reported, and the 95% confidence intervals are displayed in the brackets. [file 12014_2021_9333_MOESM8_ESM.docx]

**ADDITIONAL TABLES**

**Tables S1, S2, and S3 can be found as supplementary excel files.**

**Table S4.** List of pathways associated with gene/proteins significantly increased in HCC explant samples that exhibited post-transplant recurrence.

| Pathway description | gene count | FDR |
| --- | --- | --- |
| Malaria | 4 | 8.61E-06 |
| Bladder cancer | 3 | 0.00035 |
| Rap1 signaling pathway | 4 | 0.000619 |
| Focal adhesion | 4 | 0.000619 |
| Renal cell carcinoma | 3 | 0.000619 |
| Pancreatic cancer | 3 | 0.000619 |
| Proteoglycans in cancer | 4 | 0.00064 |
| Cytokine-cytokine receptor interaction | 4 | 0.000994 |
| ECM-receptor interaction | 3 | 0.000994 |
| HIF-1 signaling pathway | 3 | 0.00155 |
| Pathways in cancer | 4 | 0.00183 |
| PI3K-Akt signaling pathway | 4 | 0.00202 |
| Histidine metabolism | 2 | 0.00385 |
| Ras signaling pathway | 3 | 0.0103 |
| Melanoma | 2 | 0.0219 |
| TGF-beta signaling pathway | 2 | 0.0255 |
| Rheumatoid arthritis | 2 | 0.0292 |

**Table S5**. List of pathways associated with genes/proteins significantly decreased in HCC explant samples that exhibited post-transplant recurrence.

| Pathway description | gene count | FDR |
| --- | --- | --- |
| Metabolism of xenobiotics by cytochrome P450 | 9 | 5.81E-16 |
| Drug metabolism - cytochrome P450 | 9 | 5.81E-16 |
| Chemical carcinogenesis | 9 | 5.81E-16 |
| Glutathione metabolism | 9 | 6.73E-16 |
| Metabolic pathways | 6 | 3.73E-10 |
| Retinol metabolism | 10 | 2.10E-06 |
| Steroid biosynthesis | 4 | 1.30E-05 |
| Tyrosine metabolism | 3 | 3.70E-05 |
| Fatty acid degradation | 3 | 0.000218 |
| Glycolysis / Gluconeogenesis | 3 | 0.000307 |
| Terpenoid backbone biosynthesis | 3 | 0.000757 |
| PI3K-Akt signaling pathway | 4 | 0.00202 |
| Histidine metabolism | 2 | 0.00385 |
| Ras signaling pathway | 3 | 0.0103 |
| Melanoma | 2 | 0.0219 |
| TGF-beta signaling pathway | 2 | 0.0255 |
| Rheumatoid arthritis | 2 | 0.0292 |
|  |  |  |

**Table S6**. Clinical characteristics of the 29 patients transplanted for HCC beyond Milan criteria with versus without recurrence post-transplant. Median values are reported, and the 95% confidence intervals are displayed in the brackets.

| Clinical Information | non recurrent(n=13) | recurrent  (n=16) |
| --- | --- | --- |
| Age | 58 (49-76) | 57 (38-72) |
| Sex | M(12); F(1) | M(14); F(2) |
| Etiology of liver disease | HBV+ (3)  HCV+ (5)  Alcohol (2)  NASH (2) | HBV+ (0)  HCV+ (2)  NASH (10)  Other (4) |
| Number of lesions | 8 (1-20) | 13 (1-100) |
| Tumor differentiation | Moderate (12)  Poor (1) | Moderate (6)  Well differentiated (1) |
| Presence of microvascular invasion on the explant | 9 | 14 |
| Presence of microvascular invasion on the explant | 1 | 1 |
| AFP (umol/L) | 13 (3-319) | 105.5 (5-1470) |
| Death | 3 | 14 |
| Overall survival  (years) | 3.2 (0.0-9.4) | 2.2 (0.5-7.4) |
| Recurrence (months) | 3.2 (0.04  9.38 | 10.5 (3-48) |
